# Using Facial Landmark Detection on Thermal Images as a Novel Prognostic Tool for Emergency Departments

**DOI:** 10.3389/frai.2022.815333

**Published:** 2022-04-29

**Authors:** Ruben Baskaran, Karim Møller, Uffe Kock Wiil, Mikkel Brabrand

**Affiliations:** ^1^Health Informatics and Technology, The Maersk Mc-Kinney Moller Institute, University of Southern Denmark, Odense, Denmark; ^2^Department of Emergency Medicine, Odense University Hospital, Odense, Denmark

**Keywords:** triage, prognosis, thermal imaging, computer vision, machine learning

## Abstract

**Introduction:**

Emergency departments (ED) at hospitals sometimes experience unexpected deterioration in patients that were assessed to be in a stable condition upon arrival. Odense University Hospital (OUH) has conducted a retrospective study to investigate the possibilities of prognostic tools that can detect these unexpected deterioration cases at an earlier stage. The study suggests that the temperature difference (gradient) between the core and the peripheral body parts can be used to detect these cases. The temperature between the patient's inner canthus (core temperature) and the tip of the nose (peripheral temperature) can be measured with a thermal camera. Based on the temperature measurement from a thermal image, a gradient value can be calculated, which can be used as an early indicator of potential deterioration.

**Problem:**

The lack of a tool to automatically calculate the gradient has prevented the ED at OUH in conducting a comprehensive prospective study on early indicators of patients at risk of deterioration. The current manual way of doing facial landmark detection on thermal images is too time consuming and not feasible as part of the daily workflow at the ED, where nurses have to triage patients within a few minutes.

**Objective:**

The objective of this study was to automate the process of calculating the gradient by developing a handheld prognostic tool that can be used by nurses for automatically performing facial landmark detection on thermal images of patients as they arrive at the ED.

**Methods:**

A systematic literature review has been conducted to investigate previous studies that have been done for applying computer vision methods on thermal images. Several meetings, interviews and field studies have been conducted with the ED at OUH in order to understand their workflow, formulate and prioritize requirements and co-design the prognostic tool.

**Results:**

The study resulted in a novel Android app that can capture a thermal image of a patient's face with a thermal camera attached to a smartphone. Within a few seconds, the app then automatically calculates the gradient to be used in the triage process. The developed tool is the first of its kind using facial landmark detection on thermal images for calculating a gradient that can serve as a novel prognostic indicator for ED patients.

## Introduction

### The Initial Workflow

The healthcare personnel at the ED examine each patient upon admission to determine the best course of treatment. On average, the ED at OUH receives 70–100 patients per day. These patients either show up at the ED with an appointment or are admitted by ambulance based on an emergency call, i.e., without any prior appointment for admission.

After the patients arrive at the ED, they are examined in order to determine the severity of their condition. The healthcare personnel only have a few minutes to triage newly admitted patients. Patients are categorized by using a color coding system (Schmidt and Wiil, 2015). Critically ill patients need to be treated first and more stable patients will have lower priority. Examination of newly admitted patients are done by measuring vital parameters and by clinical observations.

**Figure 1:**
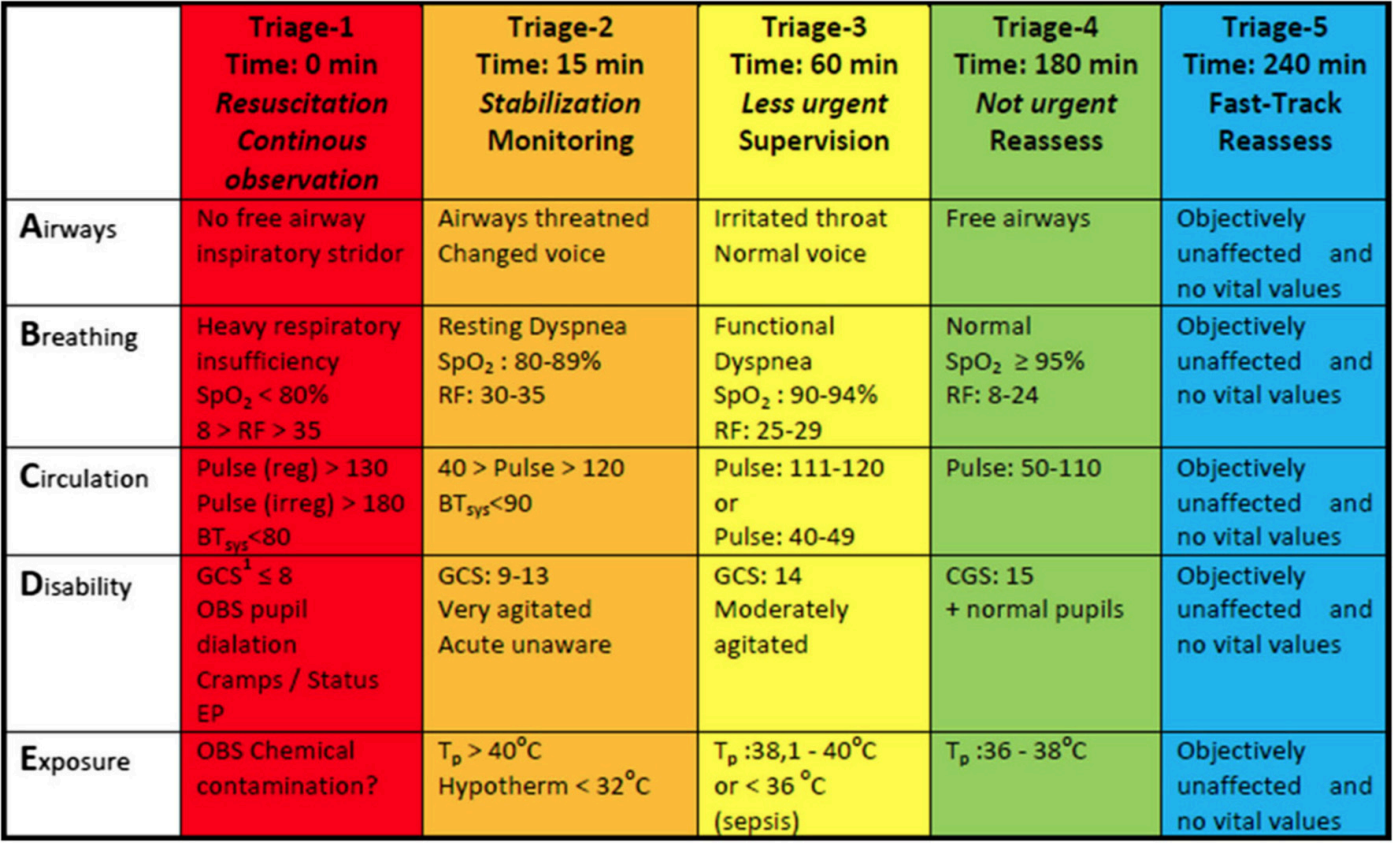
Overview of the parameters that are used to triage the patients (Schmidt and Wiil, 2015).

As seen in Figure 1, this color scale includes red, orange, yellow, green and blue. If a patient is categorized with the color “red” or “orange” it means that the patient needs urgent treatment immediately. On the contrary, if the patient is categorized with the color “green” or “blue” it means that the patient has a good prognosis and can be treated within 4 h.

### The Initial Problem

Despite this well-established examination method, there are still cases where some patients deteriorate shortly after being categorized as stable (Henriksen et al., 2014). The ED experiences that this triage system can be misleading as the vital parameters sometimes do not provide any early indicators of potential deterioration risk in the near future. A study shows that 31% of patients that are originally categorized as either “yellow”, “green” or “blue”, i.e., non-critical vital signs, deteriorate within 24 h of admission (Henriksen et al., 2014). Furthermore, it has been shown that these patients have a four times higher risk of dying within 30 days (Henriksen et al., 2014).

### The Initial Analysis

OUH has conducted a retrospective study to investigate early indicators of patients at risk of deterioration in the near future, even though the patients have normal vital signs upon admission (Henriksen et al., 2014). Research suggests that the temperature difference between the body's peripheral temperature and the core temperature can be used as an early indicator of potential deterioration even in patients that are initially assessed as being stable (Holm et al., 2018; Jensen et al., 2021a,b). It is known that the human body centralizes its blood circulation around the vital organs during critical illness (Jensen et al., 2021a,b). The blood supply and temperature around peripheral parts of the body are then reduced. This can be observed as lower temperatures on hands, feet and nose. To measure the core temperature of the body, the inner canthus can be used as a close indicator (Holm et al., 2018). Research has been conducted to investigate the most optimal reference point for measuring the peripheral temperature. The research concluded that the tip of the nose was the most accurate reference point when compared with the third finger and the ear lobes (Holm et al., 2018; Jensen et al., 2021a).

### The Initial Solution

OUH has investigated the usage of thermal images for non-invasively measuring the gradient (Holm et al., 2018; Jensen et al., 2021a,b). A thermal camera can be used for capturing the heat signature of the face in order to calculate the gradient. Unlike a normal camera that captures visible light, a thermal camera captures thermal radiation. Thermal radiation is emitted by all physical objects and matter. By capturing thermal images of newly admitted patients, it is possible to get an early indication of patients that are at risk of deterioration. This is possible by finding the temperature difference between the inner canthus (representing the core temperature) and the tip of the nose (representing the peripheral temperature). If the tip of the nose has a much lower temperature than the inner canthus, this is an indicator that blood circulation is centralizing around the vital organs and the patient may be at risk of deterioration.

### The Problem in the Initial Solution

According to the studies at OUH, the method of capturing thermal images of newly admitted patients for determining their prognosis is promising. The challenge is that it is not feasible to manually calculate the gradient as part of the daily workflow; this is simply too time consuming. Hence, there is a need for automating the method to enable it to become part of the daily workflow.

The overall research question of this study paper was defined as follows: *How can the process of capturing a thermal image and calculating the gradient between the core temperature and the peripheral temperature be automated?*

### Overview of Proposed Solution

**Figure 2:**
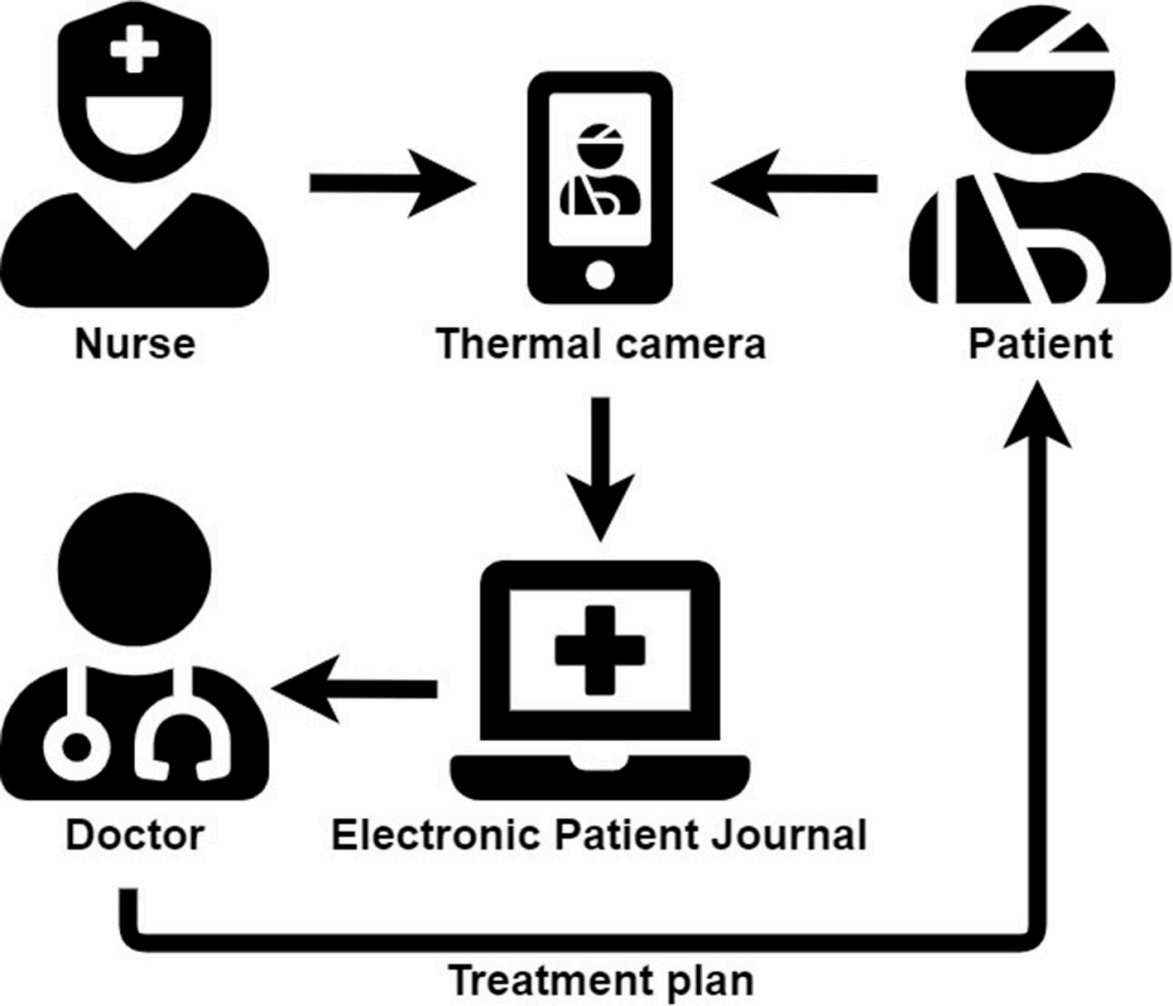
Overview of the proposed solution. Source: https://fontawesome.com/search?m=free.

Figure 2 provides an overview of the proposed solution. The first step is when the patient arrives at the ED and the nurse takes a thermal picture of the patient. The gradient is calculated and the picture and the associated gradient is submitted to the electronic patient journal. The nurse uses the gradient as part of the triage together with other available information as mentioned. Lastly, the doctor determines an appropriate treatment plan for the patient.

### Previous Work

An initial pilot study was done to examine the feasibility of the proposed work (Mohamed, 2019). This study focused on the initial stages of whether thermography could be used as a prognostic tool for detecting deterioration in ED patients. It was also investigated whether deep learning could be used for automating the process of identifying patients at risk of deterioration. The study concluded that there is a potential in using thermography along with deep learning for this purpose.

The main contribution of this study is the development of a novel prognostic tool to be used as part of the triage to identify high-risk patients.

The remainder of the paper is organized as follows. Section Related Work reviews related work. The methods used in the study are described in Section Methods. The developed solution is presented in Section Android Application, while Section Test describes the tests that were performed. Section Discussion discusses the findings of the study and Section Conclusion concludes the paper.

## Related Work

A systematic literature review was done in two parts. The first part focused on deep learning algorithms and the second part focused on open-source computer vision libraries.

**Figure 3:**
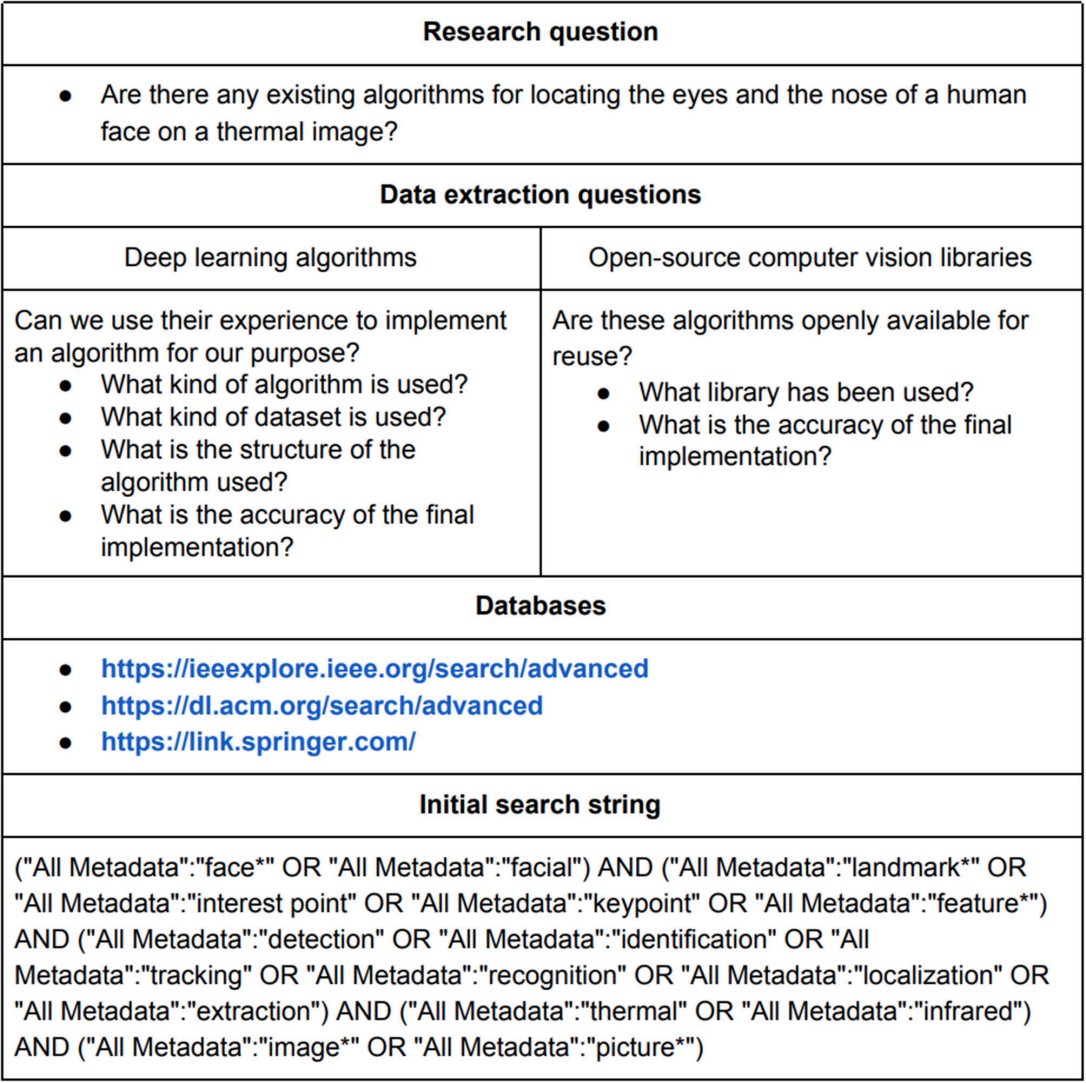
Overview of setup for systematic literature review.

Figure 3 is an overview of how the systematic literature review was set up. A main research question was defined, and based on this a search string was formed. The search string was then applied on three different scientific databases.

**Figure 4:**
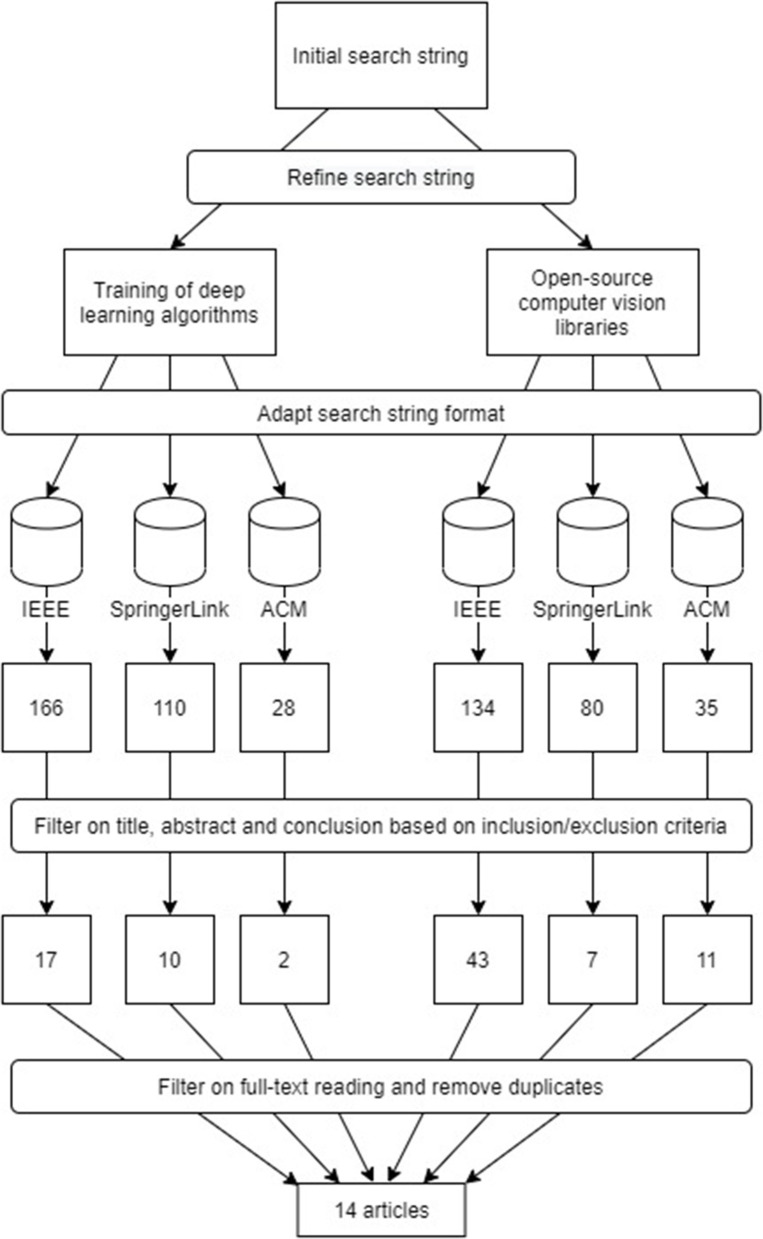
Overview of filtering process and results from systematic literature review.

Figure 4 is an overview of the process from refining and applying the initial search string on the three different databases to identifying the 14 most useful scientific papers.

After the systematic literature review, the final 14 articles were divided into three categories, based on their technical approaches; “*Deep learning algorithms*”, “*Mapping from visual image to thermal image*” and “*Extracting context from hot spots in thermal images*”.

### Deep Learning Algorithms

Several deep learning algorithms already exist for detecting facial landmarks on normal (visual) images. The question is whether these algorithms can be applied for detecting the same landmarks on thermal images. Several studies have trained deep learning algorithms with thermal images and proven that they can be successfully applied for detecting facial landmarks on thermal images.

#### A Thermal Infrared Face Database With Facial Landmarks and Emotion Labels

This paper has investigated whether providing a large number of thermal images would allow existing facial landmark detection algorithms used for normal images, to work on thermal images. The authors of the paper have created a thermal image database with more than 3,000 images of a handful of people's faces. The paper concludes that they have been successful in achieving their goal (Kopaczka et al., 2019).

#### Sobriety Testing Based on Thermal Infrared Images Using Convolutional Neural Networks

This paper has investigated the use of convolutional neural networks (CNNs) for identifying whether a person is drunk or sober. The paper concludes that using thermal images and deep learning to identify sobriety is a feasible concept (Kamath et al., 2018).

#### Thermal Facial Landmark Detection by Deep Multi-Task Learning

This paper investigates the use of deep learning for facial landmark detection and emotion recognition on thermal images of faces. The paper uses a deep learning algorithm called U-Net. The paper concludes that a robust performance has been achieved using this method (Chu and Liu, 2019).

#### Deep Features Class Activation Map for Thermal Face Detection and Tracking

This study investigates the use of CNNs for face detection on low resolution thermal images. The study concludes that this is certainly possible and that it can be used for face tracking in video data as well (Kwaśniewska et al., 2017).

### Mapping From Visual Image to Thermal Image

Highly accurate facial landmark detection algorithms already exist for normal images. These can be used to first process the normal image followed by mapping the coordinates to a corresponding thermal image. In order for the mapping to be precise, it requires that both the thermal image and the normal image are captured simultaneously at identical angles and distances.

#### Multi-Metric Evaluation of Thermal-to-Visual Face Recognition

This study has investigated the concept of converting thermal images to synthesized visible images in order to run existing computer vision algorithms on these. This method is named cross-spectrum synthesis and is defined as the practice of generating images in a desired domain using information from another domain. Their study unfortunately did not result in a high accuracy on the synthesized images (Lai and Yanushkevich, 2019).

#### Skin Temperature Extraction Using Facial Landmark Detection and Thermal Imaging for Comfort Assessment

This study has a setup with both a normal camera and a thermal camera. Since the two cameras are fixed relative to each other, the offset between the normal camera and the thermal camera can be measured and then used to align the two images. This enables the facial landmarks from the normal image to be transferred to the thermal image (Ashrant Aryal Burcin and Becerik-Gerber, 2019).

### Extracting Context From Hot Spots in Thermal Images

The human body has some specific temperature characteristics. Based on these characteristics it is possible to make assumptions about the content located at and around the hot spots in thermal images. It is known that the inner canthus will most likely be the hottest spot on the face and the warm human subject will be in clear contrast to a cold background wall. These facts can be used to locate faces and facial landmarks on a thermal image. Several studies have tried to detect eyes on thermal images. A common successful approach has been to locate hot spots in the thermal image, and associating these spots with the inner canthus. This is based on the fact that the eyes can be expected to have the highest temperature on the face. By using the eye as the reference point, studies have located other landmarks on the face. For instance, by finding the middle point between the eyes, it can be expected that the nose should be below this point.

#### Fast Eye Localization From Thermal Images Using Neural Networks

This study has investigated how to do eye localization on thermal images. The study builds on the fact that the eyes have the highest brightness in the face area, but also points out that this does not necessarily mean that the single highest pixel value in the image is equal to the eye area (Marzec et al., 2016).

#### A Comparison of Thermal Image Descriptors for Face Analysis

This study investigates the tasks of face recognition and facial expression recognition on thermal images. The study points out that thermal images are well suited for face detection and face recognition since thermal images have a low sensitivity to environment lighting conditions as well as to the skin color of the subject. The study further points out that the hottest zone in the human face is the region between the eyes and above the bridge of the nose (Carrapiço et al., 2015).

#### Automatic Eyes Localization in Thermal Images for Temperature Measurement in Cattle

This study investigates the use of thermography for doing non-invasive body temperature measurements of cattle by automatically detecting the eye region in thermal images. The study points out that by measuring the body temperature from the eye region this can be used to detect diseases and inflammation in animals (Jaddoa et al., 2018).

#### Eyes' Corners Detection in Infrared Images for Real-Time Non-contact Respiration Rate Monitoring

This study investigates the use of thermography for non-contact respiration rate monitoring. The study detects the eyes and nose by finding the highest temperature regions on the face. From these regions, the nose and the nostril is detected. The respiration is then monitored by observing the nostril area. The temperature at that area will change for each time the subject is inhaling and exhaling (Alkali et al., 2014).

#### Tracking Human Face Features in Thermal Images for Respiration Monitoring

This study also investigates the use of thermography for non-contact respiration rate monitoring. The study focuses on tracking the skin area around the tip of the nose. This is also based on the fact that the skin temperature will change in this area during respiration. The region of interest (ROI) is located by finding the warmest and coldest facial points. The study tested the method on thermal video and successfully applied it for monitoring the respiration rate (Al-Khalidi et al., 2010).

#### Concealed Knowledge Identification Using Facial Thermal Imaging

This study investigates the use of thermography for non-contact lie detection. The study uses temperature changes in the human face for detecting when a subject is lying. The study concludes that the method achieves similar or better recognition accuracy than traditional intrusive polygraph and EEG methods. The study detects the face in the thermal image by measuring the temperature difference between the subject's warm face and the cooler background (Jain et al., 2012).

#### Real-Time Facial Feature Tracking in Poor Quality Thermal Imagery

This study investigates how to do face tracking in low quality thermal images. The study looks for the mean value of pixel intensities in different areas of the face in order to do facial landmark detection (Kwaśniewska and Rumiński, 2016).

#### Respiratory Rate Estimation Using Nostril Detection in Thermal Video Streams

This study investigates how to detect the nostrils on a face by locating the area with the highest variation in temperature over time. This is based on the fact that the temperature at the nostrils will change depending on whether the person is breathing in or out. This solution can be used when thermal video is available (Mocanu et al., 2017).

### Summary

The systematic literature review gave the inspiration for exploring four different solutions: “*CNN*”, “*CNN with transfer learning*”, “*RGB-Thermal Mapping*” and “*Max-Min Template*”. These will be described further in the following section.

## Methods

This section presents how the related work summarized in the previous section gave the inspiration for the four algorithms that have been implemented in the Android application.

### CNN and CNN With Transfer Learning

#### Category: Deep Learning Algorithms

Several studies have successfully used deep learning algorithms for thermal imaging analysis. Hence this was an obvious approach to try out in this study as well. The amount of data used for training a deep learning algorithm, e.g., CNN, plays a significant role in the final performance and structure of the deep learning model. This project is only based on a relatively small dataset of 569 images, while deep learning models are typically trained with hundreds of thousands of images. Hence there is a need for using data augmentation. Another method for compensating for the small dataset, would be to use transfer learning.

**Figure 5:**
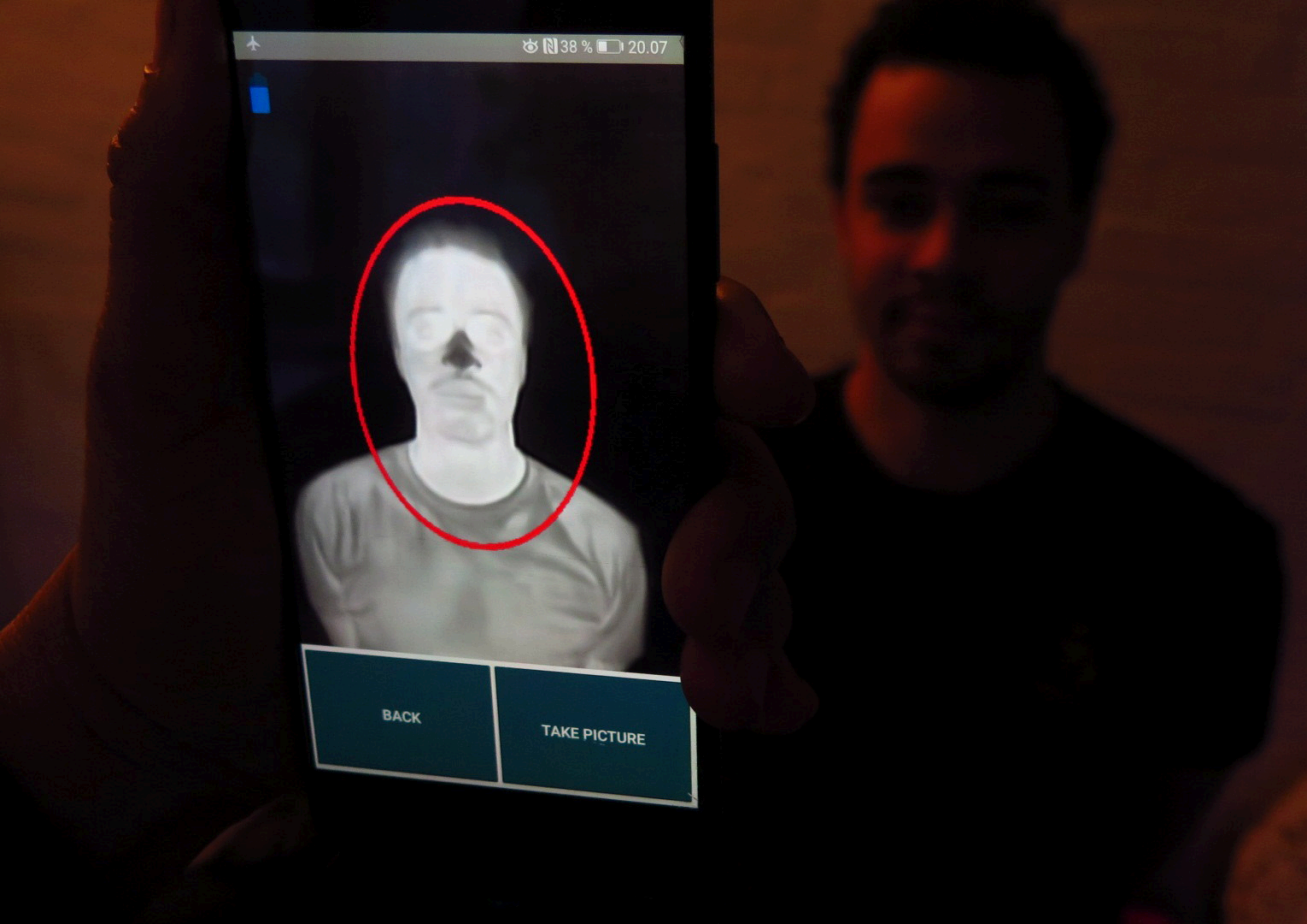
Idea behind the CNN algorithms.

Figure 5 shows the idea, where the head of the patient should be placed inside the ROI template.

Two possible solutions have been developed based on the CNN structure. One is based on the traditional CNN, and the other is based on using CNN together with transfer learning in the hope of achieving higher accuracy. Both of the algorithms will use data augmentation in order to enlarge the dataset to train on. The architecture of the developed traditional CNN is inspired by an existing open source facial landmark detection CNN algorithm (Yin, 2022). The pre-trained model used for the CNN with transfer learning algorithm is the Inception v3 (Google, 2022).

### RGB-Thermal Mapping

#### Category: Mapping From Visual Image to Thermal Image

Another approach used in some of the studies is to make the visual and the thermal image work together. Here the goal is to utilize the high performance of existing deep learning models for normal images. The idea is to first find the facial landmarks on the normal image and then transfer these locations to the thermal image.

**Figure 6:**
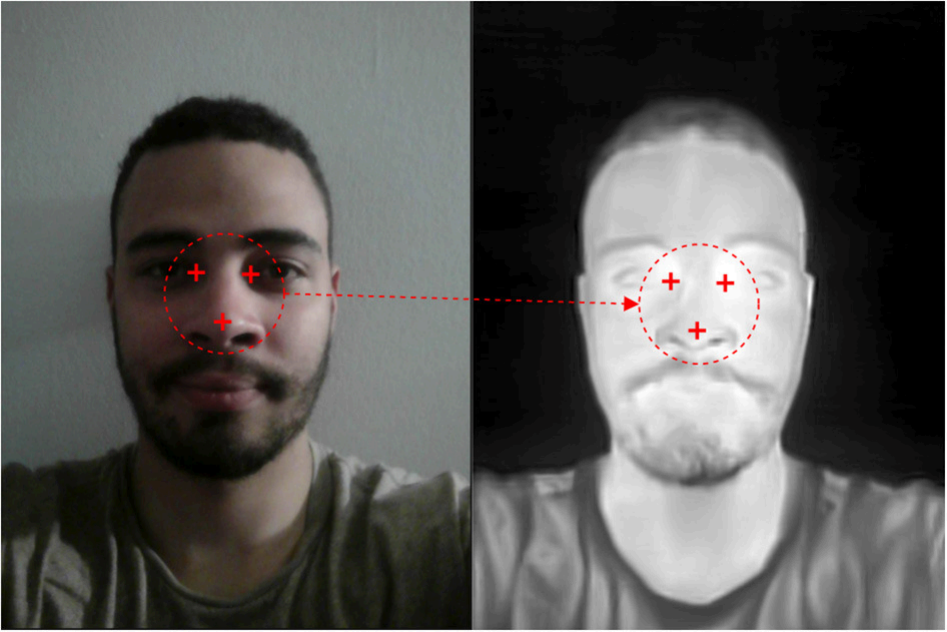
Idea behind the RGB-thermal mapping algorithm.

This requires that the images should be almost identical, i.e., the human subject should be placed at the exact same spot in the images and the images should be taken at the exact same time. An illustration of how this algorithm works is presented in Figure 6.

This is the idea behind the third solution. One important thing to consider when mapping the coordinates is that there would be a slight offset between the pictures, since the camera lenses are placed approximately one centimeter from each other in the current setup.

### Max-Min Template

#### Category: Extracting Context From Hot Spots in Thermal Images

**Figure 7:**
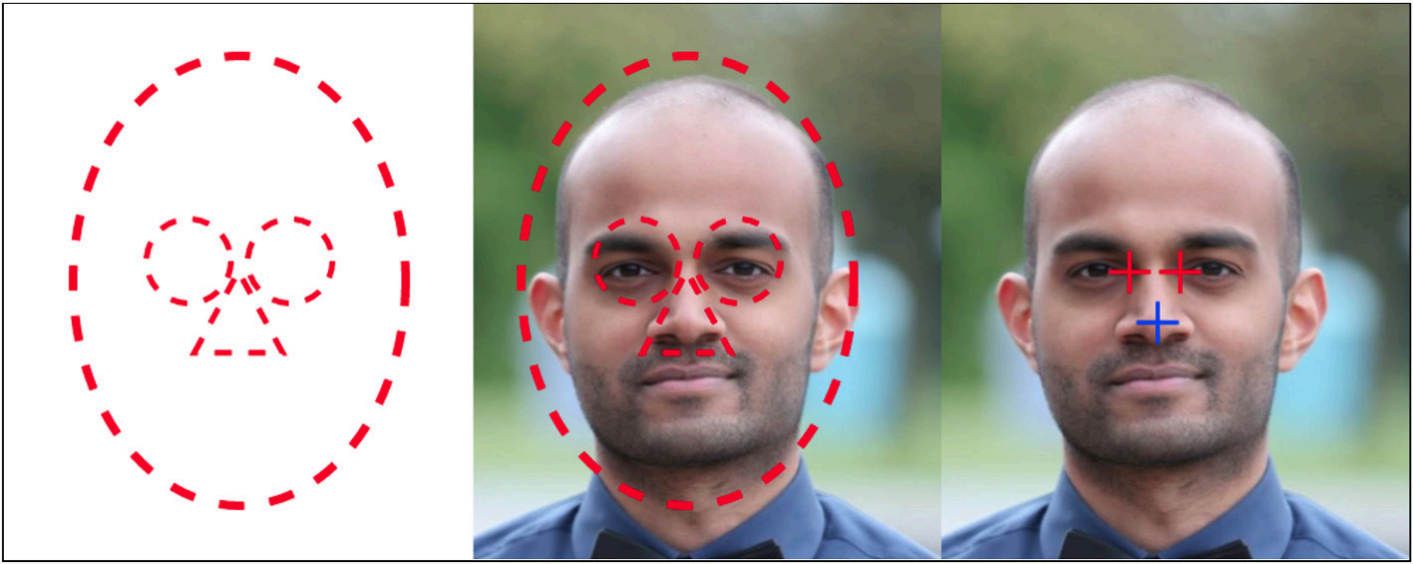
Idea behind the max-min template algorithm.

The final idea for the fourth solution is based on the fact that the human body has certain temperature characteristics that are obvious in the thermal image. Several studies have shown that the eye area is the hottest spot on a thermal image of a face and that the nose area is colder. By applying an eye-nose template when taking the picture, it is possible to pinpoint the exact area of where the eyes and the nose are placed. An example of the eye-nose template is presented in Figure 7.

This will be necessary when searching for the hottest and coldest spots inside the eye and nose areas. This fourth solution does not use any machine learning (ML), but is purely based on the fact that the hottest spot in the face is the eye, and the coldest spot below the eyes is the tip of the nose. By using this method, it is possible to locate the inner canthus and the tip of the nose in a thermal image quickly and easily.

## Android Application

Several meetings were held with the product owner (clinical professor at the ED, OUH) in order to define the requirements for the Android application. During these meetings mock-ups were presented by the team in order for the product owner to better prioritize the requirements. This section will describe the structure and design of the final product.

The hardware setup consists of an Android smartphone that has a thermal camera attached to it through its USB-C port.

**Figure 8:**
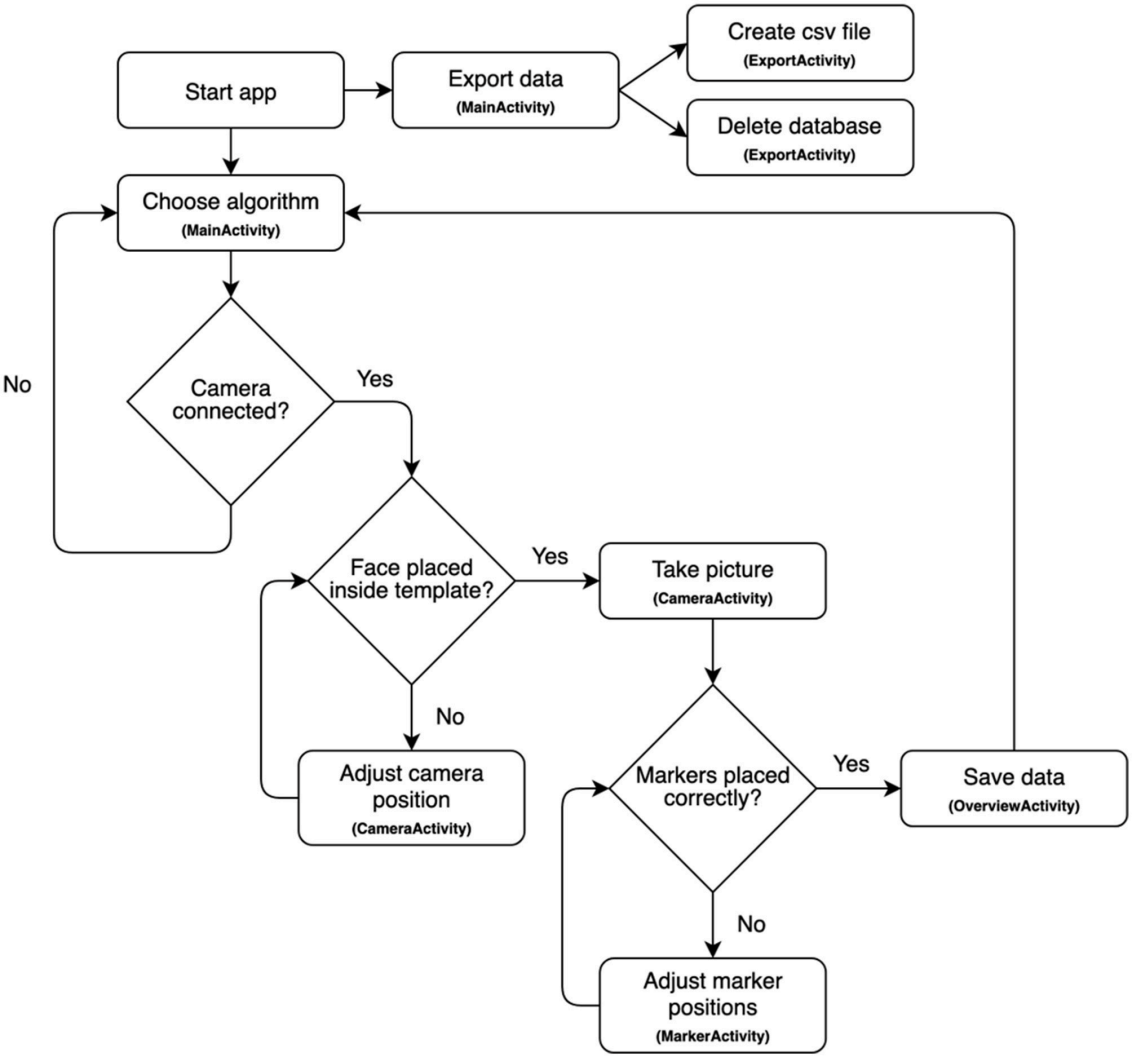
Flow chart overview of the android application.

Figure 8 shows the flow through the application. The UI-flow focuses on ensuring that even an inexperienced user can navigate the application quickly and easily.

**Figure 9:**
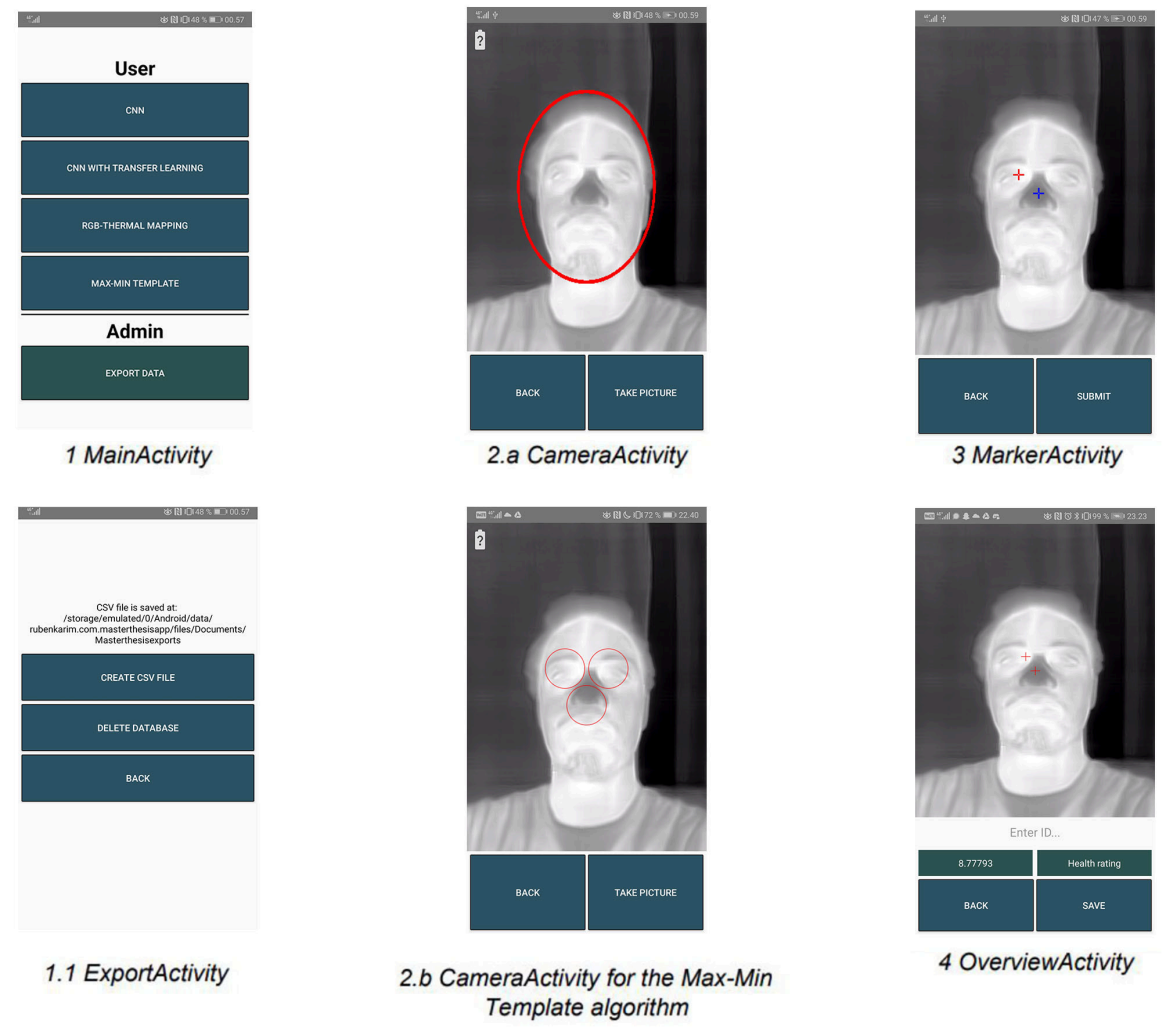
Overview of all the views in the android application.

Figure 9 shows an overview of all the views in the application along with their corresponding view names.

### MainActivity

When opening the app, the user is presented with a main menu for choosing whether to capture an image using one of the four algorithms or to export the captured images along with their data to a csv file (Figure 9.1).

### CameraActivity

This view shows the guiding face templates for the camera view. The template in view Figure 9–2.a is shown to ensure a reasonable distance between the thermal camera and the subject. This is important because the further away the subject is from the camera the more blurred the ROI area becomes. A low resolution of this area can give a poor temperature measurement since it then becomes an average of the surrounding pixel area (Vardasca, 2017). Figure 9–2.b shows a different template that is used to guide the user when using the Max-Min Template algorithm. This view also contains a power indicator for the thermal camera that can be seen in the upper left corner in screenshot Figures 9–2.a,b.

### MarkerActivity

This view shows the two markers, which are the red and blue crosses that can be moved manually by the user if needed. The red marker is used to mark the hot inner canthus and the blue marker is used to mark the cold tip of the nose. These are used for retrieving the coordinates of the ROIs that are then used for calculating the gradient (Figure 9.3).

### OverviewActivity

This view presents the results of a patient scanning. The gradient is presented to the left just above the buttons and a health rating to the right. The health rating is a color code system that can give the user an indication of the result's severity (Figure 9.4).

### Future Work

An export function has been implemented in the application, in order for the doctor to be able to export all the saved data from the smartphone to the computer. In the future, this data should instead automatically be saved directly in the electronic patient journal or patient logistics system.

### Summary

The development phase resulted in a well functioning Android application, which is ready to be used at the ED. For testing purposes a Flir One Pro camera was used.

## Test

A 5-day test phase of the Android application was conducted at OUH with the assistance of their ED healthcare personnel. The test was performed with at least one day to test each of the four algorithms. The goal of testing the application was to get feedback on the UI, investigate the precision of the algorithms and to collect suggestions for further improvements of the application. Interviews were also arranged after the test in order to gather qualitative feedback.

### Interviews

Two nurses were interviewed. The nurses had different prior knowledge regarding the project. One of the nurses had tested the application and the other one knew nothing about the project prior to the interview. As the interviews were with nurses that had two different approaches to the project, the team prepared two different sets of interview questions in order to gain as much valuable feedback as possible. The nurse that had tested the application prior to the interview was asked whether there was anything that could improve the application. The nurse that did not have any prior knowledge went through a usability test by using a simple think-aloud method. This nurse was introduced to the purpose of the application and where it was intended to be used. The nurse was then handed the application.

### Test Protocol

To ensure that the result of the test would be useful a test protocol was created. The protocol described how to use the application, and a survey was attached to get quantitative test feedback from the users.

The survey consisted of the following questions that could be rated from 1 (bad) to 5 (good) for each algorithm:

- How easy was it to place the patient's face inside the camera template overlay?
- How quickly did the algorithm find the eye and the nose?
- How precise did the algorithm place the markers on the eye and the nose?
- How easy was it to manually adjust the markers to the correct positions, in case they were initially misplaced?
- How easy was it to use the application?
- What can be improved to make the application more user friendly?

To measure the performance of the algorithms a csv file containing the ROI coordinates traced whether the markers had been manually adjusted. To measure the stability of the application any crash event was logged.

### Survey Findings

The survey results from the test were inconclusive and did not provide any useful information. The survey was inconclusive because they were not fully completed. The survey was also suffering from being incoherent as the four received answers were given by four different nurses that each had used one of the four different algorithms. The rating for each of the algorithms was therefore not comparable. One of the nurses wrote the following four useful notes which ended up being the main takeaway from the survey:

- *The Flir One Pro is too easily accidentally detached from the smartphone*.
- *The thermal lens is easily covered by accident*.
- *The Flir One Pro is connecting too slow*.
- *The FPS (frames per second) is low*.

Using a survey was in this case the wrong tool because the test only included four participants. These participants did not answer all the questions. After the interview it became clear that the participants were more inclined to talk about their experience with the application rather than writing it down in the survey form.

### Testing of Algorithms

At the end of the test week, the nurses had tested each of the four algorithms. The algorithms had been tested on a total of 62 patients at the ED. If a given algorithm correctly detected both the inner canthus and the nose tip, then the nurse would not have to adjust the markers manually in order to place them at the correct locations. In this way, the performance of each algorithm was measured. When few manual adjustments are required for a given algorithm, it means that this algorithm is performing well. Table 1 shows the statistics for how many times the markers were manually adjusted by the nurse for each test of each algorithm.

**Table 1:** Results from testing the algorithms.

**Algorithm**	**Number of tests (patients)**	**Number of times markers were manually adjusted**	**Ranking of performance (where 1 is the best)**
CNN	17	16 out of 17 times (94%)	4
CNN with transfer learning	13	12 out of 13 times (92%)	3
RGB-thermal mapping	20	1 out of 20 times (5%)	1
Max-min template	12	2 out of 12 times (17%)	2

**Figure 10:**
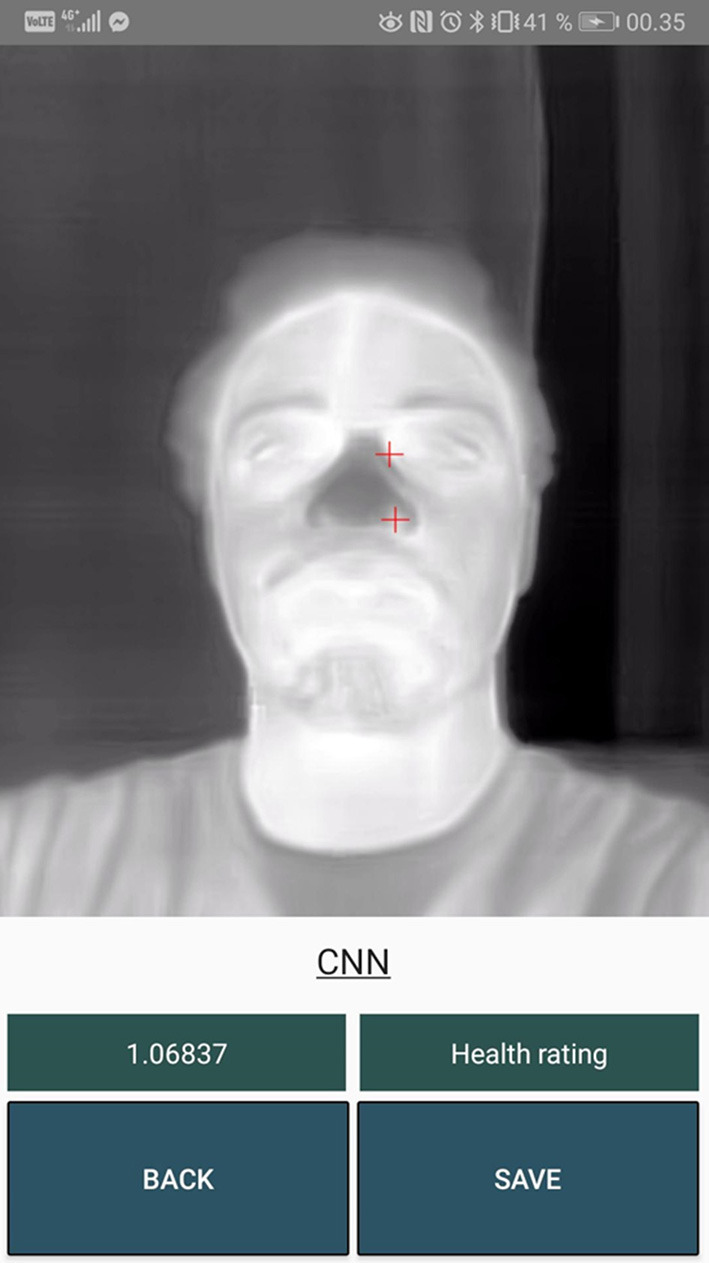
Output markers for the CNN algorithm.

**Figure 11:**
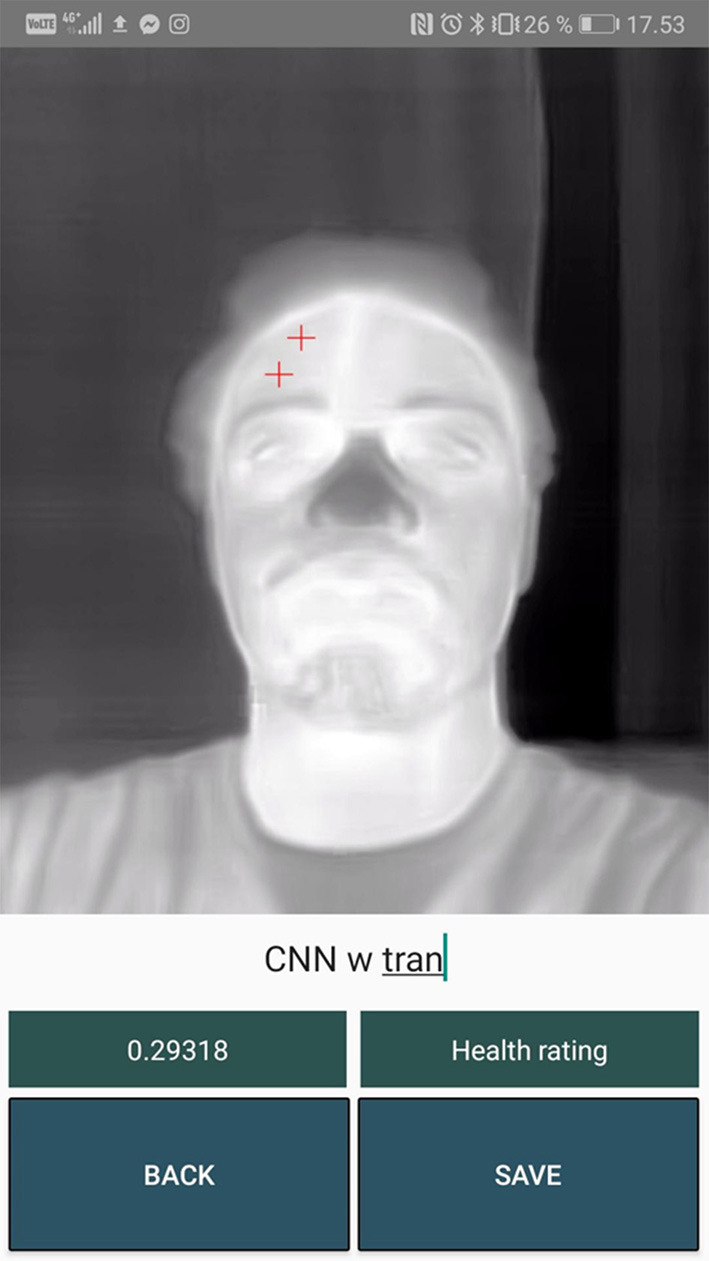
Output markers for the CNN with transfer learning algorithm.

**Figure 12:**
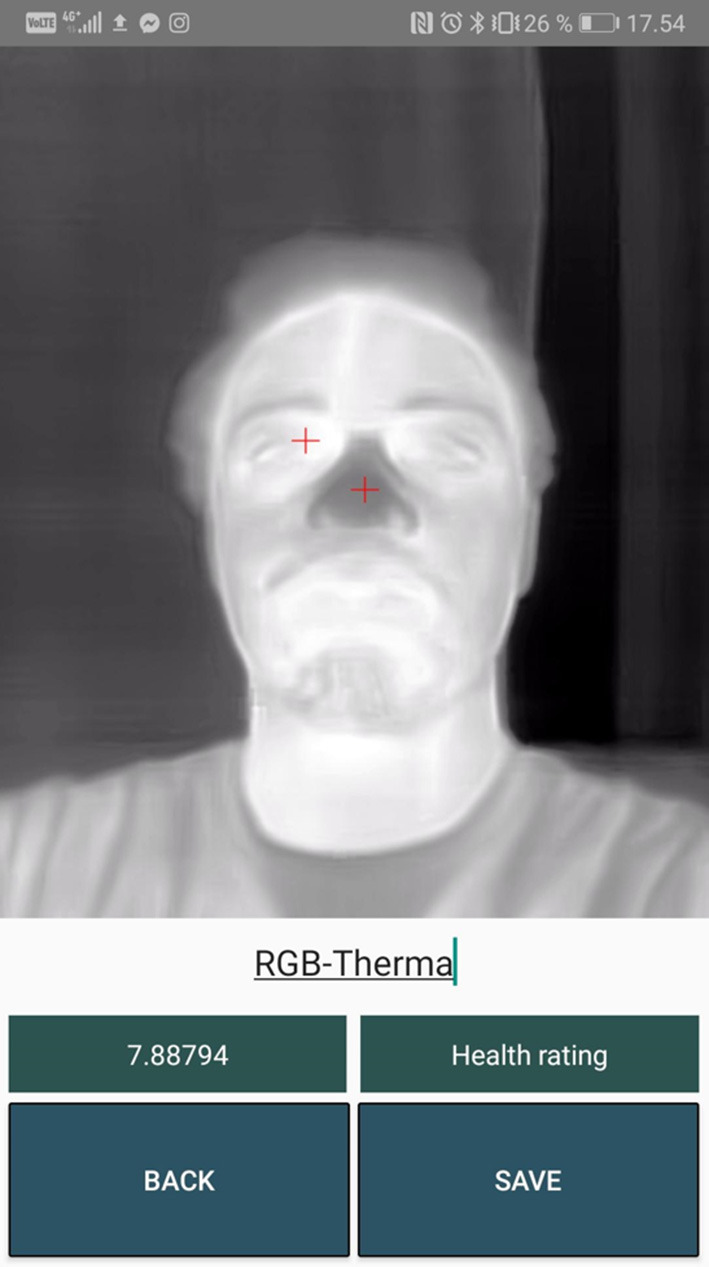
Output markers for the RGB-Thermal Mapping algorithm.

**Figure 13:**
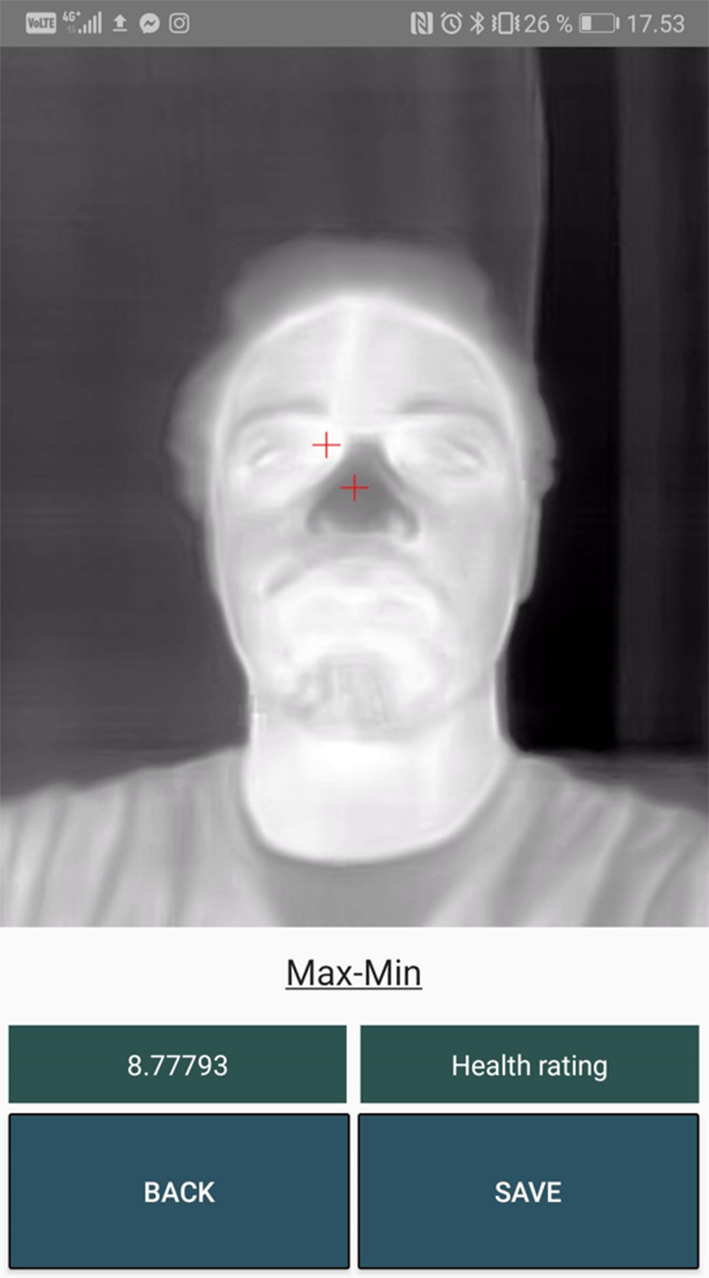
Output markers for the Max-Min Template algorithm.

Figures 10–**13** show a sample for each of the algorithms. The red markers show the coordinates that each of the algorithms returned for the inner canthus and nose tip.

### Summary

After the test phase had ended it was obvious that the RGB-Thermal Mapping algorithm would be the best algorithm to use for the final application. When the need for an angle-independent algorithm arises, a new CNN can be trained with a larger dataset that consists of the images that have been captured using the RGB-Thermal Mapping algorithm.

## Discussion

### CNN and CNN With Transfer Learning

The two CNN models performed badly. Almost every predicted marker position was misplaced and afterwards adjusted by the user as seen in Table 1. These algorithms could benefit from being trained on a much larger dataset. In Figures 10, 11 it can be seen that the markers calculated from the CNN algorithms are further away from the inner canthus and nose tip compared to the RGB-Thermal Mapping algorithm (Figure 12) and the Max-Min Template algorithm (Figure 13). Some studies have had success with using other types of algorithms for detecting facial landmarks directly on thermal images. One study has used the GentleBoost algorithm for solving the task (Martinez et al., 2010). Another study has had success with detecting landmarks on thermal images by using an open source library developed for detecting facial landmarks on normal images (Ferrari et al., 2020). A third study has developed an algorithm that uses a combination of the template-matching, knowledge-based and morphological methods for detecting facial landmarks on thermal images (Budzan and Wyzgolik, 2013). It would be worthwhile to investigate these approaches further as an alternative to the CNN approach.

### RGB-Thermal Mapping

The RGB-Thermal Mapping algorithm performed well at setting the markers at the correct positions. This was the algorithm that performed the best with a precision of 95% as shown in Table 1. The RGB-Thermal Mapping algorithm is dependent on the angle between the camera and the subject. The algorithm produces useful predictions as long as the image is captured at a fairly straight angle to the subject and the subject is kept inside the face template overlay. The challenge with this algorithm is that it is dependent on having both the normal camera and the thermal camera. Furthermore, this algorithm will not work well in low light environments. These challenges could be mitigated by applying the method of creating synthetic visible images from their corresponding thermal images. Then facial landmark detection algorithms trained on normal images could be used for finding the inner canthus and the nose tip on the synthetic image. The location of these findings could then be mapped to the thermal image in order to calculate the gradient. This method is worth investigating for future development (Di et al., 2021).

### Max-Min Template

The Max-Min Template algorithm performed reasonably well on finding the ROI locations. The Max-Min Template algorithm also produces useful predictions but through its usage it was discovered that it is not as consistent as the RGB-Thermal Mapping algorithm. This algorithm also requires more time to place the subject in the correct spot because of the three ROI areas in the face template overlay. The feedback from the nurses on this specific algorithm was regarding the difficulty to place a face in the three-circled face template overlay.

### Android Application

The overall feedback from both the survey and the interviews was that the FPS on the camera was too low; it was therefore difficult to place the patient's face in the face template overlays. This is mostly because the users are used to the 4K resolution with 30 or 60 FPS from the normal camera on a smartphone. The Flir One Pro camera updates with <9 FPS which is very low compared to the normal camera. Flir One Pro is developed in this way because of an export law from the U.S. Department of Commerce which states that exporting a thermal camera with a FPS equal to or larger than nine requires a license issued by the U.S. authorities (FLIR, 2022). Further feedback was that the red and blue markers on the MarkerActivity view were too small. The end users had trouble manually placing these markers on the ROI locations. A way to mitigate this would be to enlarge the markers or to enable zooming on the image. It could also be mitigated by implementing a way for selecting a marker and moving it by following the relative movement of a touch and drag anywhere on the screen. Lastly the end users would like to flip the phone 180 degrees since this felt more natural to them, and it mitigates the risk of covering the camera lenses with their hands.

### Weather Conditions

Another challenge that needs to be addressed is to determine if the weather is affecting the temperature of the patient's face too much which could cause the gradient to become misleading. This could for instance happen if the patient walks straight into the ED after being outside in the freezing cold (Brabrand et al., 2021).

### Summary

The algorithms were evaluated by the team and the 5-day testing period at OUH gave great insights into the performance of each of the four algorithms. It was obvious that the two CNN algorithms could be improved significantly. The two other algorithms did satisfy the needs of the user, but the low FPS of the thermal camera had a negative impact on the overall user experience. The best performing algorithm was the RGB-Thermal Mapping. While the answers from the survey were of questionable quality, the face-to-face interviews with the nurses were very valuable. Multiple new suggestions for improving the application were gathered during these interviews.

## Conclusion

The main problem statement has been addressed and the objective has been accomplished by developing an Android application that can capture a thermal image of a patient's face and then automatically calculate and show the gradient between the inner canthus and the tip of the nose.

The systematic literature review gave important insights and inspiration for how to develop algorithms for facial landmark detection on thermal images. It was not possible to train a highly accurate CNN model from scratch in order to apply it for facial landmark detection on thermal images. The CNN algorithm as well as the CNN with transfer learning algorithm did not end up with a satisfying accuracy. The Max-Min Template algorithm ended up with a relatively satisfying algorithm, but the algorithm that met the need of the end user was the RGB-Thermal Mapping algorithm. For the RGB-Thermal Mapping algorithm, a pre-trained CNN model from Firebase ML kit was used for doing facial landmark detection on a normal image.

The output coordinates from this model were then successfully mapped to the corresponding thermal image, which was captured simultaneously with the normal image.

The main contribution of this study is a novel solution that automates the process of finding the temperature gradient between a patient's core temperature and peripheral temperature as a prognostic indicator identifying high-risk patients. The developed solution makes it possible to integrate the process of using the temperature gradient in the ED workflow. Another study has identified the untapped potential of using computer vision and machine learning for solving healthcare related issues (Leo et al., 2020). With our study, the healthcare sector moves one step closer to realizing that potential.

A prospective study is currently being planned as future work. The study will be conducted at the ED at OUH to test the effects of using the developed prognostic tool as part of the triage process. If the prospective study has positive results, then the system can be integrated into the ED workflow as a permanent part of the triaging process.

## Data Availability Statement

The datasets presented in this article are not readily available due to patient confidentiality.

## Ethics Statement

Ethical review and approval was not required for the current study in accordance with the local legislation and institutional requirements. The patients/participants provided their written informed consent to participate in this study. Written informed consent was obtained from the individuals for the publication of any potentially identifiable images or data included in this article.

## Author Contributions

RB and KM developed the presented prognostic tool in collaboration with UW and MB. All authors contributed to the writing of the manuscript. All authors contributed to the article and approved the submitted version.

## Conflict of Interest

The authors declare that the research was conducted in the absence of any commercial or financial relationships that could be construed as a potential conflict of interest.

## Publisher's Note

All claims expressed in this article are solely those of the authors and do not necessarily represent those of their affiliated organizations, or those of the publisher, the editors and the reviewers. Any product that may be evaluated in this article, or claim that may be made by its manufacturer, is not guaranteed or endorsed by the publisher.
